# Final follicular maturation by administration of GnRH agonist plus HCG versus HCG in normal responders in ART cycles: An RCT

**Published:** 2017-07

**Authors:** Maryam Eftekhar, Maryam Farid Mojtahedi, Sepideh Miraj, Malihe Omid

**Affiliations:** 1 *Reasearch and Clinical Center for Infertility, Yazd Reproductive Sciences Institute, Shahid Sadoughi University of Medical Sciences, Yazd, Iran.*; 2 *Recurrent Abortion Research Center, Yazd Reproductive Sciences Institute, Shahid Sadoughi University of Medical Sciences, Yazd, Iran.*; 3 *Department of Obstetrics and Gynecology, Endocrinology and Female Infertility Unit, Roointan Arash Women’s Health Research and Educational Hospital, Tehran University of Medical Sciences, Tehran, Iran.*; 4 *Department of Obstetrics and Gynecology, School of Medicine, Shahrekord University of Medical Sciences, Shahrekord, Iran.*

**Keywords:** Trigger, Gonadotropin-releasing hormone, Agonist, Antagonist, Normal responder, Human chorionic gonadotropin

## Abstract

**Background:**

Gonadotropin-releasing hormone agonists (GnRH-a) was increasingly used for triggering oocyte maturationfor the prevention of ovarian hyperstimulation syndrome. Studies suggest that GnRH-a might be used as a better trigger agent since it causes both Luteinizing hormone and follicle stimulating hormone release from a physiologic natural cycle.

**Objective:**

The aim of this study was to evaluate the effect of dual-triggering in assisted reproductive technology outcomes.

**Materials and Methods:**

192 normal responder women aged ≤42 years and 18< Body Mass Index <30 kg/m^2 ^enrolled in this single-blind randomized controlled trial. All participants received antagonist protocol. For final triggering, women randomly were divided into two groups. Group, I was triggered by 6500 IU human chorionic gonadotropin (hCG) alone, and group II by 6500 IU hCG plus 0.2 mg of triptorelin. The implantation, chemical, clinical and ongoing pregnancy, and abortion rates were measured.

**Results:**

The mean of retrieved oocytes and obtained embryos were statistically higher in the dual-trigger group (group I), but the implantation and pregnancy rates were similar in two groups.

**Conclusion:**

The results of our study did not confirm the favorable effect of dual-triggered oocyte maturation with a GnRH-a and a standard dosage of hCG as an effective strategy to optimize pregnancy outcome for normal responders in GnRH-antagonist cycles. We think that this new concept requires more studies before becoming a universal controlled ovarian hyperstimulation protocol in in vitro fertilization practice.

## Introduction

Reducing the rate of sever ovarian hyperstimulation syndrome (OHSS) is one of the ideal goals of controlled ovarian hyperstimulation (COH) in assisted reproduction technology (ART) programs (1, 2). Gonadotropin-releasing hormone agonists (GnRH-a) was used for triggering oocyte maturation for OHSS prevention increasingly. Initially, Gonen *et al* suggested that GnRH-a might be used as a better trigger since it causes both follicle stimulating hormone (FSH) and Luteinizing hormone (LH) release from a physiologic natural cycle (3). Several studies reported that GnRH-a decreased the implantation rate while it increased the abortion rate in comparison with human chorionic gonadotropin (hCG). This is probability attributed the luteal phase deficiency and decreased endometrial receptivity. 

Therefore, modified luteal phase supports estradiol, progesterone and also a low dose of hCG either at oocyte retrieval or during the luteal phase was proposed in these cycles (3-9). The fact that gene expression pattern and downstream LH signaling receptors is different between hCG and GnRH-a triggered patients made several investigators to study the effect of co-administration of GnRH-a and hCG triggering to improve ART outcomes (4-6). Several studies showed significant improvement in in vitro fertilization (IVF) outcomes when a dual trigger was used without a significant increase in OHSS rate in high responders (6-9). Simultaneous administration of a standard dose of hCG and GnRH-a for triggering the final oocyte maturation has been studied in a limited number of studies (6-9).

Schachter *et al* initially proposed the dual trigger concept in normal responders (8). The investigators hypothesized that GnRH antagonist binding to endometrial GnRH receptors stimulates post-receptor events that reduces implantation rate. They suggested that administering preovulatory GnRH-a displaces bound GnRH antagonist from endometrial GnRH receptors, so that the blocked receptors will be activated and subsequent pre-implantation post-receptor events will be enabled. They demonstrated significantly improved ongoing pregnancy rate in the group who received hCG and GnRH-a for triggering. The other possible explanation in GnRH agonist-triggered cycles is the induction of FSH surge in addition to LH surge, which has been postulated to be instrumental in resumption of the oocyte’s meiotic processes, conferring some advantages for embryos development (8).

Regarding this promising results, we studied whether dual triggering for final oocyte maturation with a single dose of GnRH-a and a standard dose of hCG could improve pregnancy outcome in GnRH-antagonist IVF/ Intracytoplasmic sperm injection (ICSI) cycles in normal responders.

## Materials and methods

All normal responder women by tubal or male infertility factor which has been referred to Yazd Research and Clinical Center for Infertility between April 2014 to February 2015 were enrolled in this single-blind randomized controlled trial.

The inclusion criteria were 18< Body mass index <30 kg/m^2^, and age ≤42 yr with the history of infertility for at least 1 yr that were candidate for ART protocol. Our exclusion criteria were the presence of endocrine disorders such as diabetes mellitus, hyperprolactinemia, thyroid disorders, congenital adrenal hyperplasia, Cushing syndrome, polycystic ovary syndrome, congenital uterine anomaliesdisorders, repeated implantation failure, day-3 FSH concentration ≥10 IU/L or serum anti-Mullerian hormone ≤1.0 ng/mL, and azoospermia (10). In the next step, high or poor response to COH were excluded.The poor ovarian response was defined as serum estradiol (E_2_) level less than 500 pg/mL on the day of triggering or the number of retrieved oocytes less than three. The high ovarian response was defined as an E_2_ level higher than 3,000 pg/mL on the day of triggering or the number of retrieved oocytes more than 15.

Those who met the inclusion criteria, started ovarian stimulation with a flexible dosage of recombinant FSH (150-225 IU) on the second day of the menstrual cycle for 5 consecutive days. Once the leading follicle had reached 13 mm, co-treatment with the GnRH antagonist 0.25 mg/day, was initiated. Gonadotropins doses were further adjusted according to vaginal ultrasound measurements of follicular diameter. When at least two leading follicles reached 17 mm in diameter, women randomly were divided into two groups for final triggering according to a computer-generated randomization table: group I was triggered by 6500 I.U. hCG alone and group II by 6500 IU hCG plus 0.2 mg of triptorelin (Decapeptyl; Ferring, Switzerland). Women with high or poor response to COH were excluded in this step. Oocyte retrievals were performed under transvaginal ultrasound guidance 34-36 hr after triggering. Embryo transfers were performed 48-72 hr after oocyte retrieval. 

The luteal phase was supported by 400 mg vaginal progesterone suppositories twice a day (Fertigest; Aburaihan pharmaceutical co., Iran) starting on the day of oocyte retrieval. Serum β-hCG was measured 14 days after embryo transfer, and a value above 50 IU/mL was considered to be a positive pregnancy. The luteal phase support was then continued until the 10^th^ wk of gestation. The primary outcome was clinical pregnancy rate and the secondary outcome was implantation rate, chemical pregnancy, ongoing pregnancy, and abortion rate.

**Power of the study**

It is assumed that dual triggering will improve clinical pregnancy rate from 29-44%. Accordingly,the power was set at 80% and it was found that 100 cycles were needed in each group to detect this difference.

**Ethical consideration**

This study protocol was approved by Yazd Research and Clinical Center for Infertility ethics committee (Reference code: 315, dated 93.4.2). A midwife not involved in the study randomized women according to the randomization table on the day of triggering final oocyte maturation and the physicians were blinded. Oral informed consent was obtained from each participant.

**Statistical analysis**

Statistical Package for the Social Sciences, version 15.0, SPSS Inc, Chicago, Illinois, USA (SPSS) was used for all statistical calculations. Chi-squared test and the sample t-test test was used for comparing categorical data. P<0.05 was considered statistically significant.

## Results

**Figure 1:**
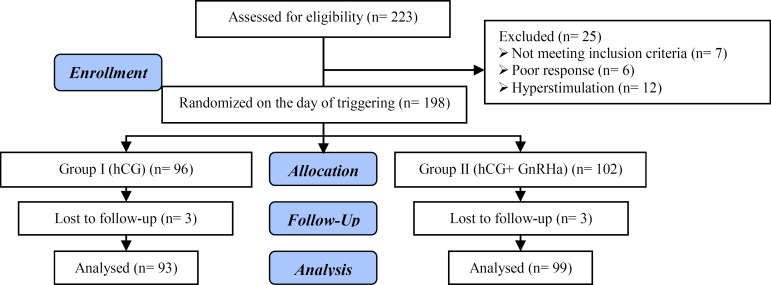
Flowchart of the study design

From 223 eligible women, totally 192 participants were enrolled in two groups (Figure 1). No significant differences were found in participants and cycles’ demographics characteristics between two groups (Table I, II). There were not statistically significant differences in the total recombinant FSH dose, duration of stimulation, duration of GnRH-antagonist treatment, serum E_2_ on the day of the trigger (Table II). 

The mean of retrieved oocytes and obtained embryos were statistically higher in the dual-trigger group (Table II), but the implantation and pregnancy rate were similar in two groups (Table III).

**Table I:** Participants Demographics characteristics of study participants

		**Group I (hCG +GnRHa) (n=99)**	**Group II (hCG)(n=93)**	**p-value**
BMI		24.13 ± 2.87	24.07 ±2.98	0.88^*^
Age		30.06 ± 5.30	30.49 ± 4.79	0.54 ^*^
Duration of infertility (yr)		6.34 ± 3.85	6.23 ± 4.09	0.70 ^*^
Day-3 FSH level (IU/L)		6.59 ± 2.76	6.14 ± 2.59	0.23 ^*^
Infertility type (%)				
	Primary	71.3	78.4	0.49**
	Secondary	28.7	21.6	

Data presented as Mean±SD.

BMI: Body Mass Index

* Independent Sample *t-*test

** Chi-Square test

**Table II:** Comparison of cycles characteristics between two study groups

	**Group I (hCG +GnRHa) (n=99)**	**Group II (hCG) (n=93)**	**p-value** ^*^
Total dose of gonadotropins (IU)	1753.81 ± 584.16	1739.33 ± 507.22	0.88
Duration of stimulation (day)	10.21 ± 1.69	10.25 ± 1.62	0.60
Estradiol on trigger day(pg/mL)	1506.01 ± 699.66	1508.20 ± 722.14	0.75
No. oocytes retrieved	10.85 ± 4.71	9.35 ± 4.35	0.009
MII retrieved oocytes	8.80 ± 3.99	7.98 ± 3.85	0.12
embryos formed in each cycle	6.86 ± 4.16	5.34 ± 3.80	0.007
transferred embryos	1.72 ± 0.86	1.66 ± 0.82	0.61

Data presented as Mean±SD.

* Independent Sample *t-*test

**Table III:** Comparison of clinical outcomes between two study groups

	**Group I (hCG +GnRHa) (n=99)**	**Group II (hCG) (n=93)**	**p-value** *
Implantation rate	11	10	0.50
Chemical pregnancy rate	(30/99) 30.3	(24/93) 25.8	0.51
Clinical pregnancy rate	(26/99) 26.3	(21/93) 22.6	0.30
Ongoing pregnancy rate	(24/99) 24.2	(20/93) 22.9	0.77
Abortion rate	(2/25) 8	(4/24) 16.7	0.35

Data presented as n (%).

* Chi-Square test

## Discussion

Our results indicate that mean number of retrieved oocytes, mature metaphase II oocytes and formed embryos were higher in the dual-trigger group compared with the hCG. However, we found no significant differences in implantation and pregnancy rates. Ovulation has been preceded by a surge of LH and FSH during the menstrual cycle which triggers the oocyte maturation process. During the IVF cycle, hCG is given to mimic the LH surge, and the oocyte retrieval is generally performed 35-37 hr later.

However, hCG has no FSH receptor activity, unlike the GnRH-a, which releases an endogenous FSH (and LH) surge. FSH plays a role in oocyte maturation during the natural menstrual cycles and may have a benefit for patients with infertility treated with IVF. FSH has been shown to be important in in vitro maturation of oocytes and to induce ovulation independent of the LH surge in animal studies. The FSH surge induces LH receptor formation on luteinized granulosa cells and promotes oocyte maturation and cumulus expansion (12).

Decleer *et al* compared 5000 I.U. hCG with a combination of 5000 I.U. hCG and GnRH-a 36 hr prior to ovum pick-up in a randomized controlled trial in normal responder women. They showed no differences in a mean number of retrieved, mature oocytes, and even pregnancy rates. Also, their study showed the number of patients who received at least one excellent quality embryo and the number of cryopreserved embryos was significantly higher in dual trigger group (13).

In contrast to our results, Ming-Huei Lin *et al* found a significant increase in mature oocytes, implantation, and clinical pregnancy rate in dual trigger group. They used the dual triggering for final oocyte maturation by combining a single dose of GnRH-agonist with a 6500 I.U. hCG. However, their results in the mean number of retrieved oocytes were similar to our results (7). 

Griffin *et al* evaluated the effect of the dual trigger (GnRHa and hCG 5,000 IU or 10,000 IU, 35-37 h) prior to oocyte retrieval in women with a previous history of >25% immature oocytes retrieved. Despite a significantly higher proportion of mature retrieved oocytes with the dual trigger, the observed IVF outcome remained poor, probably due to patients underlying oocyte dysfunction (12).

GnRH has been suggested to play multiple roles in the endometrial receptivity regulation and embryo implantation (14, 15). Not surprisingly, concerns have been raised regarding the impact of GnRH-antagonist exposure during the preimplantation stage (16). GnRH has been shown to modulate matrix metalloproteinases in the placental trophoblasts that mediate trophoblast cell invasion and extracellular matrix degradation (17, 18). 

Rackow *et al* demonstrated that the expression of endometrial HOXA10, a modulator of endometrial receptivity, was significantly decreased in endometrial stromal cells of GnRH-antagonist IVF cycles when compared with either natural or GnRH-agonist IVF cycles (19). In a review, Orvieto evaluated the studies published about the different type of administration of GnRH-a in combination with hCG for final follicular maturation, aimed to clarify which is the best method for triggering. the review concluded that there was a comparable or even better oocyte/ embryos quality in patients, not at risk of OHSS with GnRH-a and hCG trigger as compared to hCG trigger. 

The article proposed GnRH-a concomitant to the standard hCG trigger dose, to improve oocyte/embryo yield and quality. It suggested that concomitant administration of GnRH-a and hCG may be offered 34-37 hr prior to oocyte retrieval (dual trigger) in normal responder patients or 40 hr and 34 hr prior to oocyte retrieval (double trigger) in women with abnormal final follicular maturation, despite normal response to induction, to improve ART outcome (20). 

## Conclusion

The results of our study did not confirm the favorable effect of dual-triggered oocyte maturation with a GnRH-agonist and a standard dosage of hCG as an effective strategy to optimize pregnancy outcome for normal responders in GnRH-antagonist cycles. We think that this new concept requires more study before becoming a universal COH protocol in IVF practice.

## note

***Registration ID in IRCT: IRCT2015031221420N2***
